# Infectious Disease: Safety Net for Malaria?

**Published:** 2007-05

**Authors:** Harvey Black

Pyrethroid-treated nets may be losing their effectiveness in preventing malaria, says a study in the February 2007 issue of *Emerging Infectious Diseases*. Mark Rowland, a senior lecturer at the London School of Hygiene and Tropical Medicine, and his colleagues found that treated nets killed only 30% of malaria-carrying *Anopheles gambiae* mosquitoes in an area of Benin where the *kdr* gene is highly prevalent; mosquitoes with this gene are less vulnerable to pyrethroids’ neurotoxicity. In an area where *kdr* is rarely found, 98% of the mosquitoes were killed. “These findings are the first clear evidence of pyrethroids’ failing to control an *An. gambiae* population that contains *kdr* resistance at high levels,” the researchers write.

Treated nets are a primary means of fighting malaria in countries where the disease is endemic. “I think what we’ve identified here is the start of a problem which is going to get worse,” says Rowland. “It may be that what we’ve uncovered at Benin exists elsewhere, but it hasn’t been studied rigorously enough to prove that point.”

WHO scientist Pierre Guillet says more study is necessary on actual malaria reduction conferred by treated nets, not just on the mortality of vectors, to draw broader conclusions about the impact of the *kdr* gene. The February paper also notes that in Côte d’Ivoire, treated nets prevented malaria regardless of the prevalence of the *kdr* gene in mosquitoes—perhaps, Rowland speculates, because the *An. gambiae* mosquitoes there may be of a different type.

The insecticide both kills and repels mosquitoes, strengthening the physical barrier of the nets, which by itself is not enough to efficiently prevent malaria in real-life situations, explains Guillet. Volunteers in the study stayed in huts typical of the sort in which inhabitants of the region live. Even though treated nets were not lethal to mosquitoes with the *kdr* gene, they did deter 44% of the resistant mosquitoes from entering the huts.

According to Frank Collins, a professor of biological sciences at the University of Notre Dame, the implications of the study “are that resistance is going to emerge.” New insecticides would not be the final answer, since mosquitoes probably would eventually develop resistance to them. This eventuality could be delayed, note Collins and Guillet, by treating nets with two insecticides, each with a different mechanism of action.

Even though such a strategy does not yet exist, and even in the face of developing resistance, treated nets remain vital, says Rowland: “Nets are still the best means of protection against malaria that we know of.” The effort to provide treated nets has become a worldwide campaign. Nothing But Nets, founded by a diverse coalition of groups including the United Methodist Church and the National Basketball Association, is collecting money to buy and distribute nets in Africa.

## Figures and Tables

**Figure:**
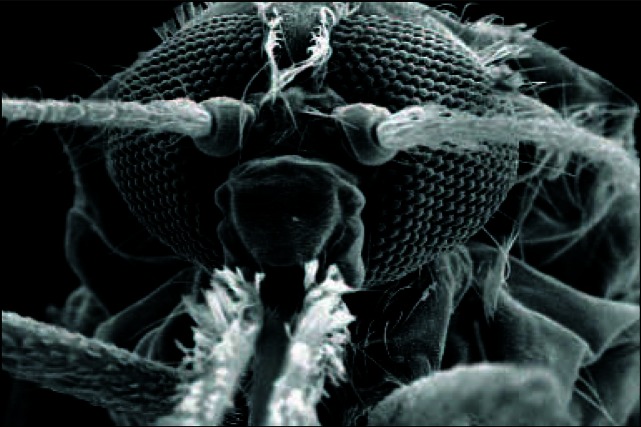
Anopheles gambiae

